# Investigation of the Impact of Hydrogen Bonding Degree in Long Single-Stranded DNA (ssDNA) Generated with Dual Rolling Circle Amplification (RCA) on the Preparation and Performance of DNA Hydrogels

**DOI:** 10.3390/bios13070755

**Published:** 2023-07-23

**Authors:** Xinyu Wang, Huiyuan Wang, Hongmin Zhang, Tianxi Yang, Bin Zhao, Juan Yan

**Affiliations:** 1 Laboratory of Quality and Safety Risk Assessment for Aquatic Products on Storage and Preservation (Shanghai), Ministry of Agriculture, Shanghai Engineering Research Center of Aquatic-Product Process & Preservation, College of Food Science and Technology, Shanghai Ocean University, Shanghai 201306, China; m210300896@st.shou.edu.cn (X.W.); d220300089@st.shou.edu.cn (H.W.); hmzhang@shou.edu.cn (H.Z.); 2 Faculty of Land and Food Systems, The University of British Columbia, Vancouver, BC V6T 1Z4, Canada; tianxi.yang@ubc.ca; 3 Department of Biochemistry and Molecular Biology, The University of British Columbia, Vancouver, BC V6T 1Z4, Canada

**Keywords:** three-dimensional network, nucleic acid material, physical cross-linking, pure DNA hydrogel, nucleic acid signal amplification technique, biosensing

## Abstract

DNA hydrogels have gained significant attention in recent years as one of the most promising functional polymer materials. To broaden their applications, it is critical to develop efficient methods for the preparation of bulk-scale DNA hydrogels with adjustable mechanical properties. Herein, we introduce a straightforward and efficient molecular design approach to producing physically pure DNA hydrogel and controlling its mechanical properties by adjusting the degree of hydrogen bonding in ultralong single-stranded DNA (ssDNA) precursors, which were generated using a dual rolling circle amplification (RCA)-based strategy. The effect of hydrogen bonding degree on the performance of DNA hydrogels was thoroughly investigated by analyzing the preparation process, morphology, rheology, microstructure, and entrapment efficiency of the hydrogels for Au nanoparticles (AuNPs)–BSA. Our results demonstrate that DNA hydrogels can be formed at 25 °C with simple vortex mixing in less than 10 s. The experimental results also indicate that a higher degree of hydrogen bonding in the precursor DNA resulted in stronger internal interaction forces, a more complex internal network of the hydrogel, a denser hydrogel, improved mechanical properties, and enhanced entrapment efficiency. This study intuitively demonstrates the effect of hydrogen bonding on the preparation and properties of DNA hydrogels. The method and results presented in this study are of great significance for improving the synthesis efficiency and economy of DNA hydrogels, enhancing and adjusting the overall quality and performance of the hydrogel, and expanding the application field of DNA hydrogels.

## 1. Introduction

Deoxyribonucleic acid (DNA) hydrogels are a promising class of macroscopic three-dimensional (3D) materials that offer a unique combination of high hydrophilicity and mechanical properties in polymer hydrogels [1] along with the remarkable biological functions of DNA such as structural designability, biocompatibility, selection specificity, molecular recognition ability, and responsiveness to environmental factors [2,3]. Because of these advantages, DNA hydrogels have wide-ranging applications in diverse fields such as food safety [4,5], medical diagnostics [6,7,8], environmental analysis [9,10], and controllable drug delivery and release [11,12]. Despite the availability of different chemical covalent linkages [13,14] and non-covalent physical cross-linking methods for preparing DNA hydrogels [15,16], the substantial cost of bulk-scale fabrication of pure DNA hydrogels and their low mechanical properties significantly limit their potential applications [1,17].

Rolling circle amplification (RCA) is a widely used isothermal nucleic acid amplification technique [18,19] that utilizes DNA polymerase to repeatedly copy a circular DNA template [20], resulting in the production of a long single-stranded DNA (ssDNA) molecule with a periodic sequence with high efficiency under mild reaction conditions [21,22]. RCA technology has attracted significant attention in the development of multifunctional assemblies for disease diagnosis and treatment, food safety testing, environmental quality monitoring, and other applications [23]. Zhao et al. provided a comprehensive overview of the fundamental engineering principles employed in the design of RCA technologies. They also discussed the latest advancements in RCA-based diagnostics and bioanalytical tools. Additionally, the authors summarized the utilization of RCA for the construction of multivalent molecular scaffolds and nanostructures, highlighting their applications in biology, diagnostics, and therapeutics [24]. In 2012, in a groundbreaking study, Luo and his colleagues developed a mechanical metamaterial made from a pure DNA hydrogel using RCA technology and multi-primed chain amplification [25]. The DNA hydrogel exhibited unusual mechanical properties, with a solid-like nature in water and a liquid-like nature after the removal of water. Since then, RCA technology has been extensively explored for the self-assembly of ultralong ssDNA to construct hydrogels with the desired functions and performance for use in various biosensing [26,27,28] and medical scenarios [29,30]. For instance, Yang et al. developed a DNA hydrogel using the double RCA assembly strategy for the separation of bone marrow stromal cells [31]. Long ssDNA generated using RCA with aptamer sequences ensured the specific anchoring of cells, while the physically cross-linked network exhibited a moderate storage modulus (G′) of about 12 Pa, which could minimize mechanical damage to cells. These studies have shown that RCA technology is a simple, rapid, and cost-effective synthetic method that can be used for the bulk production of DNA hydrogels, and the functionalization of hydrogels can be achieved by programming the circular template sequences and integrating different DNA bio-functional modules (e.g., aptamer, G-quadruplex, i-motif structure) [32,33,34,35]. However, regulatory analysis of the mechanical properties of pure DNA hydrogels made with RCA technology has not been thoroughly explored.

In fact, the integration of various nanomaterials, including gold/silver nanoparticles (Au/Ag NPs), carbon materials, magnetic nanoparticles, and clays, into DNA hydrogels [36,37] has become a commonly used approach to regulating the properties of DNA hydrogels [38]. This is attributed to the fact that nanomaterials offer a broader spectrum of opportunities and potential for the utilization of DNA hydrogels, as they enhance mechanical properties, improve stability, regulate morphology and structure, and provide responsiveness. The mechanical properties of the hydrogel are largely determined by the concentration and quantity ratio of the components used in the hydrogel preparation. While this strategy is highly promising, it is important to note that the introduction of nanomaterials may negatively impact the biocompatibility and biodegradability of DNA hydrogels, and the presence of selected nanomaterials may interfere with hydrogel matrix cross-linking. In addition, there have been reports of toughening hydrogels in response to stimulation using heat, light, pH, and salt [39], but the precise control of these stimuli is also a challenge [40].

It is widely recognized that a DNA strand comprises a phosphate–deoxyribose backbone and one of four bases: adenine (A), guanine (G), cytosine (C), or thymine (T). Moreover, single-stranded DNA (ssDNA) can form stable double-stranded DNA (dsDNA) structures through hydrogen bonds between A and T and between C and G, which is known as “Watson–Crick base pairing” [41]. Hydrogen bonding, as a dynamic and weak non-covalent bond, is a significant form of physical cross-linking in addition to chain entanglement, hydrophobic interaction, and other interactions. It plays a pivotal role in the interaction between DNA molecules and is the primary driving force for DNA strand hybridization [18]. Because of its dynamic nature and degree of bonding, hydrogen bonding provides great flexibility in regulating molecular structure and properties. Hence, it is imperative to investigate the relationship between the degree of hydrogen bonding, the preparation of DNA hydrogels, and the resulting mechanical properties of DNA hydrogels. However, there is a dearth of relevant reports on this topic, highlighting the need for further research in this area.

In this study, we have experimentally demonstrated the influence of hydrogen bonding on the preparation and performance of DNA hydrogels. This study involves the utilization of ssDNA generated using dual-RCA technology as precursors for the preparation of DNA hydrogels. The degree of hydrogen bonding between the ssDNA precursors was regulated by designing circular template sequences. The entrapment efficiency for AuNPs varied in DNA hydrogels prepared with different degrees of hydrogen bonding, which reflects the differing mechanical properties of the prepared DNA hydrogels. Our findings not only present a novel approach to developing DNA hydrogels with distinct properties but also establish a foundation for their potential applications in diverse fields.

## 2. Materials and Methods

### 2.1. Materials

All synthetic DNA sequences were tabulated as shown in Table 1. DNA sequences were synthesized and HPLC purified by Shenggong Biotechnology Co., Ltd. (Shanghai, China). T4 DNA ligase (1000 U/μL), phi29 DNA polymerase (5 KU), deoxynucleotides (dNTP, 10 mM) were from Sangon Biotechnology Co., Ltd. (Shanghai, China). Tetrachloroauric acid hydrate (HAuCl_4_·3H_2_O) was purchased from J&K Scientific (Shanghai, China). Bovine serum albumin (BSA) was purchased from Strem Chemicals, Inc., Massachusetts USA. In addition, 50× TAE buffer (2 M Tris-HCl, 100 mM EDTA, pH 8.4), 4S GelRed and 4S GelGreen nucleic acid staining agents (10,000× aqueous solution), sodium citrate (C_6_H_5_Na_3_O_7_·2H_2_O), and other reagents and chemicals were purchased from Shenggong Biotechnology (Shanghai) Co., Ltd. and were at least analytical-reagent grade. All aqueous solutions were prepared using Milli-Q water (18.2 MΩ·cm).

### 2.2. Apparatus

Constant temperature oscillation metal bath (HCM100-Pro) and handheld Centrifuge (D1008) were provided by Dalong Xingchuang Experimental Instrument Co., Ltd. (Beijing, China). Vortex mixer (Mixer 4K, Shenggong Biotechnology Co., Ltd., Shanghai, China); fluorescence microscope (CKX-41, Olympus, Tokyo, Japan); S-3400-emission scanning electron microscope (Hitachi, Tokyo, Japan); Haake Mars rheometer (Thermo Fisher, Karlsruhe, Germany); UV–visible spectrophotometer (UV-2540, Shimazu Co., Kyoto, Japan); gel imaging and analysis system (Bio-Rad Co., Ltd., Hercules, American).

### 2.3. Preparation of the Circular Templates

A volume of 5 μL of 100 μM phosphorylated linear DNA was mixed with 10 μL of 100 μM primer and annealed at 90 °C for 5 min. The solution was then slowly cooled to room temperature. Next, 2 μL of T4 ligase (1000 U/μL) and 3 μL of 10× T4 ligase buffer were added to the reaction mixture, which was mixed well and incubated overnight at 25 °C. The T4 ligase was subsequently heat-inactivated at 65 °C for 10 min, after which the prepared circular DNA template solution was stored at 4 °C.

### 2.4. Preparation of the ssDNA Precursors Using Dual Rolling Circle Amplification (RCA)

Amplification was carried out in a 60 μL reaction system containing 4 μL of circular DNA template solution, 1 μL of phi29 polymerase (5 KU), 5 μL of phi29 polymerase buffer (10×), 5 μL of dNTPs (10 mM), and 45 μL of TE buffer (1×). The reaction mixture was thoroughly mixed and then incubated at 37 °C for 3 h, resulting in the generation of ssDNA products. In parallel, another RCA reaction system was performed using a circular template with a different DNA sequence, which also produced an ssDNA product. These two kinds of ssDNA products, having different DNA composition sequences, were used as precursors for the subsequent preparation of DNA hydrogel.

### 2.5. Preparation of DNA Hydrogels with ssDNA Precursors

Two types of ssDNA precursors with different sequences (60 μL) were mixed together thoroughly for 10 s at 25 °C, resulting in the formation of a visible DNA hydrogel.

### 2.6. Preparation of BSA-Coated Au Nanoparticles (AuNPs–BSA)

Colloidal gold nanoparticles (AuNPs) with an average diameter of 30 nm were prepared using the conventional Frens method [42]. Specifically, 50 mL of Milli-Q water and 50 μL of 10% HAuCl4 solution were added to a 250 mL round-bottom flask equipped with a reflux device and heated to boiling. Then, 1% trisodium citrate (0.7 mL) was rapidly added with vigorous stirring for 30 min [43]. During this process, the colorless solution gradually changed to a purplish-red color. The heating was stopped, and the solution was stirred overnight at room temperature. Finally, the prepared AuNPs solution was filtered through a filter membrane with a pore size of 0.22 μm and stored at 4 °C.

To prepare the AuNPs–BSA compound, 1 mL of 30 nm AuNPs and 200 μL of 30% BSA (diluted with Milli-Q water) were mixed well and incubated overnight at room temperature. The mixture was then centrifuged at 4 °C, 11,000 r/min for 20 min. The supernatant was discarded, and the precipitate was resuspended in 1 mL of 1× PBS solution. This centrifugation step was repeated three times to remove any free BSA. Finally, the precipitate was resuspended in 100 μL of 1× PBS, and the obtained AuNPs–BSA compound was stored in the dark at 4 °C.

### 2.7. Characterization Methods

Rheological tests: Rheological experiments were conducted on DNA hydrogel samples using the time scan mode. Storage modulus (G′) and loss modulus (G″) values were measured at a fixed frequency (1 Hz) and fixed strain (1%) at 25 °C. Time-scan rheological tests were also performed using a 20 mm parallel-plate geometry (gap size 0.01 mm) at a fixed frequency (1 Hz) and strain (1%) at 25 °C for 3 min.

Scanning electron microscope (SEM) imaging: Different DNA hydrogel samples were flashed frozen in liquid nitrogen and further freeze-dried for at least 48 h. The microstructures of these samples were then sputtered with gold and studied under a scanning electron microscope at a voltage of 5 kV.

UV–visible spectroscopy characterization: The UV–visible absorption spectral measurements were conducted using a UV-2540 spectrometer. The UV absorbance of 20 µL of supernatant AuNPs–BSA samples from three distinct DNA hydrogels was measured at 520 nm.

## 3. Results

### 3.1. Mechanism of the Degree of Hydrogen Bonding Based on Dual RCA Strategy for Regulating the Mechanical Properties of DNA Hydrogels

**Scheme 1:**
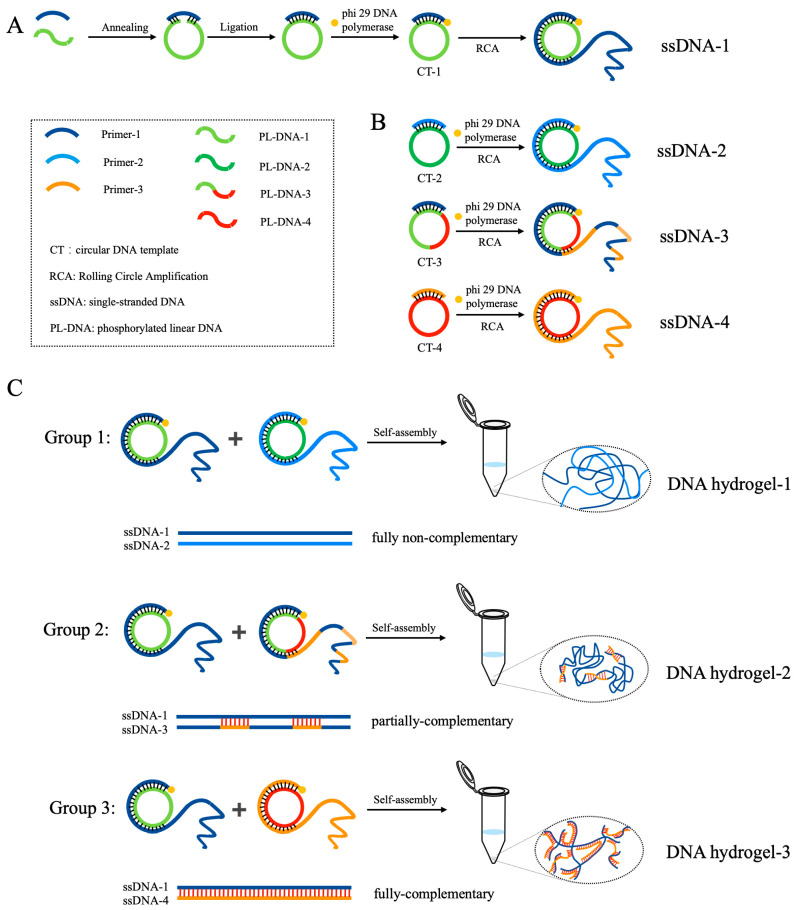
Schematic illustration of DNA hydrogels prepared using ultralong single-stranded DNA (ssDNA) with varying hydrogen bonding degrees generated with dual rolling circle amplification (RCA). (**A**): Preparation of circular DNA template (CT-1) and generation of ssDNA chain (ssDNA-1) by RCA reaction. (**B**): Generation of three ssDNA chains (ssDNA-2, ssDNA-3, and ssDNA-4) by RCA reaction based on three types of circular DNA templates (CT-2, CT-3, and CT-4). (**C**): Group 1 shows DNA hydrogel-1 prepared after the self-assembly of two fully non-complementary DNA strands, ssDNA-1 and ssDNA-2; group 2 shows DNA hydrogel-2 prepared after the self-assembly of two partially-complementary DNA strands, ssDNA-1 and ssDNA-3; group 3 shows DNA hydrogel-3 prepared after the self-assembly of two fully-complementary DNA strands, ssDNA-1 and ssDNA-4.

The schematic illustration of the investigation of the effect of hydrogen bonding degree on the performance of DNA hydrogels prepared with dual RCA technology is shown in Scheme 1. The method comprises three major steps: preparation of four types of circular DNA templates (CT-1, CT-2, CT-3, and CT-4), followed by four independent but simultaneous RCA reactions in the presence of phi29 DNA polymerase that generate four ultralong ssDNA chains with repeated periodic sequences complementary to the circular DNA template (ssDNA-1, ssDNA-2, ssDNA-3, and ssDNA-4). By programming the circular template sequences, ssDNA-1 is expected to construct varied degrees of hydrogen bonding with the other three chains (ssDNA-2, ssDNA-3, and ssDNA-4). Specifically, ssDNA-1 is completely non-complementary to ssDNA-2, partially complementary to ssDNA-3, and fully complementary to ssDNA-4. In the final step, those three groups of DNA strands (ssDNA-1 + ssDNA-2, ssDNA-1 + ssDNA-3, and ssDNA-1 + ssDNA-4) with different degrees of hydrogen bonding were used as precursors and mixed in one test tube to prepare three groups of DNA hydrogels (DNA hydrogel-1, DNA hydrogel-2, and DNA hydrogel-3) through self-assembly. The performance of three sets of DNA hydrogels, prepared using ultralong ssDNA chains with different degrees of hydrogen bonding in accordance with the dual RCA strategy, was compared and analyzed.

### 3.2. Preparation and Characterization of RCA Products

**Figure 1:**
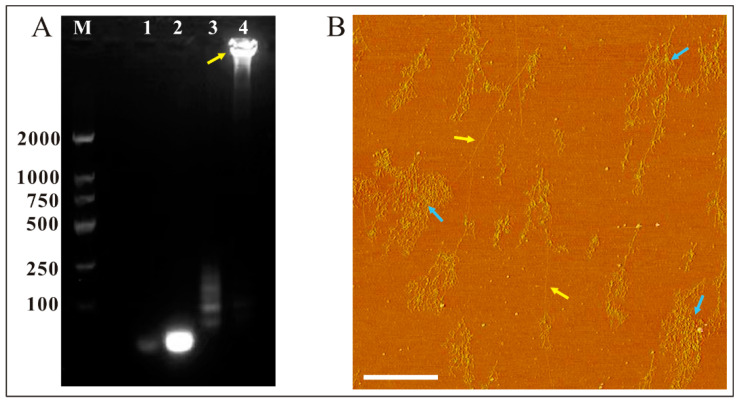
(**A**): Agarose gel electrophoresis results of the RCA product. M: DNA marker; 1: primer-1; 2: phosphorylated linear DNA-1 (PL-DNA-1); 3: circular DNA template-1 (CT-1); 4: RCA products (ssDNA-1). (**B**): AFM phase image of RCA products. Yellow arrows indicate single-stranded DNA and blue arrows indicate nanostructures by DNA random coiling. The scale bar represents 1 μm.

The successful formation of DNA hydrogels relies on the efficacy of the RCA reaction. To validate the feasibility of the study, agarose gel electrophoresis was utilized to characterize the RCA products, as shown in Figure 1A. The circular DNA template (CT) was composed of a phosphorylated linear single-stranded DNA (PL-DNA) and another single-stranded DNA serving as a linker. The linker DNA had complementary ends to the linear DNA, which brought the 5′ end and 3′ end of the linear DNA closer together, thereby forming a phosphodiester bond under the action of T4 ligase and obtaining a ligated circular DNA [44]. In addition, the linear DNA sequence was used as the RCA reaction template, and the linker DNA was used as the RCA primer. The primer was extended along the CT with the aid of phi29 DNA polymerase to produce tandem repeated sequences complementary to the CT. The formation of ligated CT-1 was verified by the gel results, where CT-1 (lane 3) migrated slower than linear DNA-1 (lane 2) and primer-1 (lane 1). Furthermore, the RCA products were trapped in the loading well (yellow arrow, lane 4) and unable to migrate downward when exposed to an electric field, indicating the large molecular weight of the products and thus the success of the RCA reaction. The ligation of other CTs and their mediated RCA were also verified to be successful (Figure S1).

The Atomic Force Microscope (AFM) was used to further characterize the RCA reaction and provide a visual representation of the structural details of the ssDNA chains. As depicted in Figure 1B, the DNA strands were observed to intertwine and form disorderly, concentrated, coiled complexes (blue arrows), instead of being fully stretched and in a single-stranded linear state (yellow arrows). This was mainly attributed to the flexibility of ssDNA, which is influenced by non-covalent forces such as electrostatic interactions, hydrophobic interaction, and intra-stranded base pairing, leading to the formation of non-specific secondary structures. The successful RCA reaction provided the precursors for DNA hydrogels and served as the foundation for their preparation.

### 3.3. Preparation and Morphological Characterization of DNA Hydrogels

**Figure 2:**
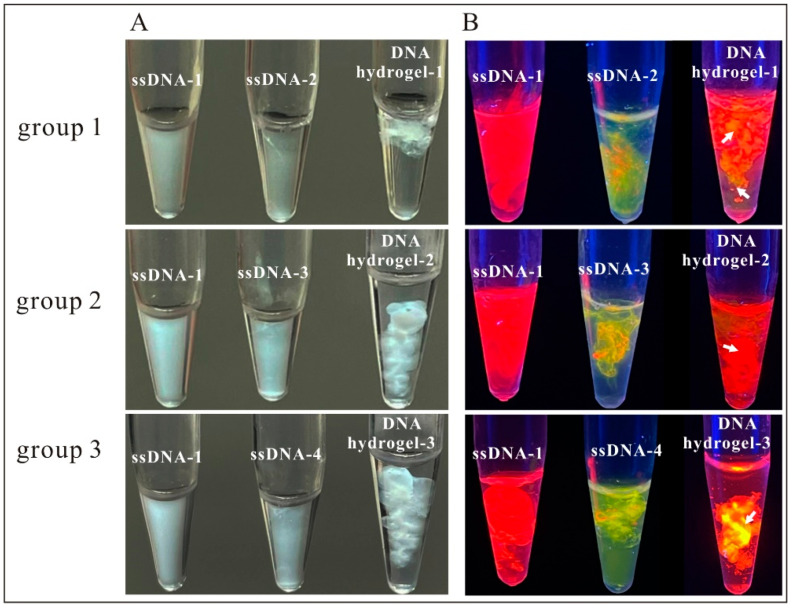
Images of DNA hydrogels prepared using assembly of ssDNAs with different degrees of hydrogen bonding under natural light (**A**) and UV light (**B**). ssDNA-1 was stained with 4sGelRed; ssDNA-2, ssDNA-3, and ssDNA-4 were stained with 4sGelGreen. Group 1 shows DNA hydrogel-1 prepared after the mixture of two completely non-complementary DNA strands, ssDNA-1 and ssDNA-2; group 2 shows DNA hydrogel-2 prepared after the mixture of two partially complementary DNA strands, ssDNA-1 and ssDNA-3; group 3 shows DNA hydrogel-3 prepared after the mixture of two fully complementary DNA strands, ssDNA-1 and ssDNA-4.

To visualize the different states of DNA molecules in solution before and after the formation of DNA hydrogel, imaging results under white light and ultraviolet (UV) modes are presented separately. Figure 2A shows two chains of ssDNA-1 and ssDNA-2, which were generated with RCA and were completely non-complementary in terms of sequence composition, mixed to produce DNA hydrogel-1 (group 1). Before mixing, the precursor DNA solution in the two centrifuge tubes appeared slightly cloudy, but no obvious precipitate was found. However, after mixing, a clear white flocculent aggregation rapidly appeared in the upper layer of the solution in the tube. Upon exposure to UV, red and green filaments were observed in the two ssDNA tubes (ssDNA-1 stained with 4sGelRed and ssDNA-2 stained with 4sGelGreen) (group 1, Figure 2B), indicating that RCA produced large amounts of ssDNA products that were not visible to the naked eye under white light. Similarly, DNA hydrogel-2 and DNA hydrogel-3 were formed, observed, and compared under white light and 365 nm UV light exposure (group 2 and group 3).

As the degree of hydrogen bonding between ssDNA-1 and its counterpart DNA increased, the resulting DNA hydrogels gradually sank to the bottom of the tube, indicating a more compact structure. This was further confirmed through fluorescence imaging. DNA hydrogel-1 appeared as a fluffy, sponge-like structure with the largest volume, while DNA hydrogel-2 resembled a twisted wool ball, and DNA hydrogel-3 displayed firm lamellar structures (right column, Figure 2B). Additionally, a comparison of fluorescence color changes after gel preparation revealed that DNA hydrogel-1 maintained a certain degree of independence between the red and green DNA strands (white arrows, group 1, Figure 2B), while DNA hydrogel-2 exhibited a color change to orange (white arrow, group 2, Figure 2B), and DNA hydrogel-3 was dominated by a yellow product (white arrow, group 3, Figure 2B). In accordance with these results, we tentatively hypothesized that non-complementary long ssDNA molecules would form a loosely structured DNA hydrogel because of the physical entanglement of ssDNA chains, while partially complementary ssDNA would form a DNA hydrogel through base pairing and physical intertwining. Fully complementary ssDNA, on the other hand, would primarily form a relatively dense hydrogel through DNA hybridization based on base pairing.

### 3.4. Characterization of Mechanical Properties and Microstructure of DNA Hydrogels

**Figure 3:**
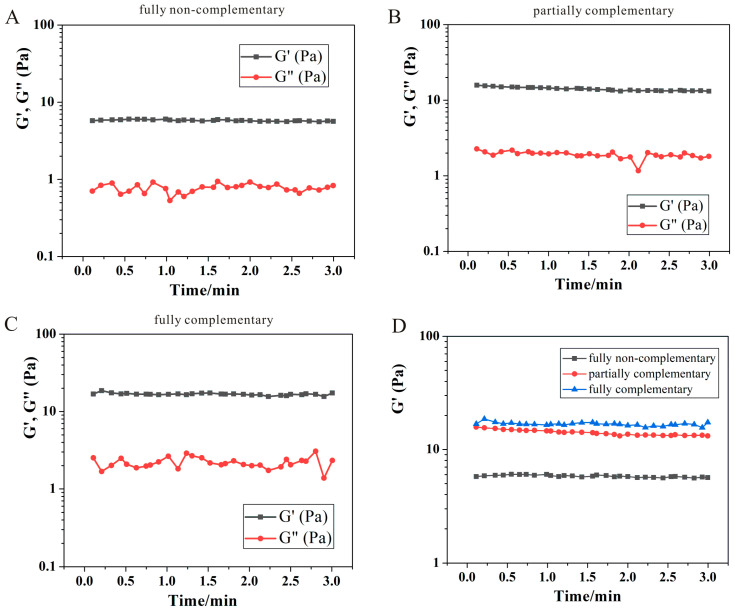
Time-scan rheological performance of DNA hydrogels prepared using assembly of ssDNAs with different degrees of hydrogen bonding. (**A**): DNA hydrogel-1 prepared with two completely non-complementary DNA strands (ssDNA-1 and ssDNA-2). (**B**): DNA hydrogel-2 prepared with two partially complementary DNA strands (ssDNA-1 and ssDNA-3). (**C**): DNA hydrogel-3 prepared with two fully complementary DNA strands (ssDNA-1 and ssDNA-4). (**D**): Comparison of the energy storage modulus (G′) of DNA hydrogels with different degrees of hydrogen bonding.

To investigate this hypothesis, the rheological characteristics of the DNA hydrogels were further examined. As illustrated in Figure 3A–C, it is evident that for the hydrogels produced from entirely non-complementary DNA chains (Figure 3A), partially complementary DNA chains (Figure 3B), and fully complementary DNA chains (Figure 3C), the storage modulus (G′) was largely higher than the loss modulus (G″), indicating the solid nature of these three types of DNA hydrogels. Furthermore, the G′ values of these hydrogels were compared, which can reflect the mechanical properties of the hydrogel. As shown in Figure 3D, the G′ value for the DNA hydrogel based on fully complementary ssDNA chains was the highest, followed by partially and non-complementary ones. Additionally, it was observed that the G′ values of gels based on fully and partially complementary ssDNA were significantly greater than that based on non-complementary ssDNA, which is consistent with the above morphology results and suggests that the mechanical properties of DNA hydrogel can be adjusted by introducing complementary base pairs into ssDNA chains. Interestingly, the G′ value of the hydrogel based on fully complementary DNA strands was not substantially larger than that of the hydrogel based on partially complementary DNA chains. This may be attributed to the hydrogel preparation method, in which two types of ssDNA were mixed with vortexing and incubated at room temperature for only 10 s. It is likely that this process was insufficient in enabling the complete hybridization of all complementary sequences in the mixture, leaving some ssDNA unpaired.

**Figure 4:**
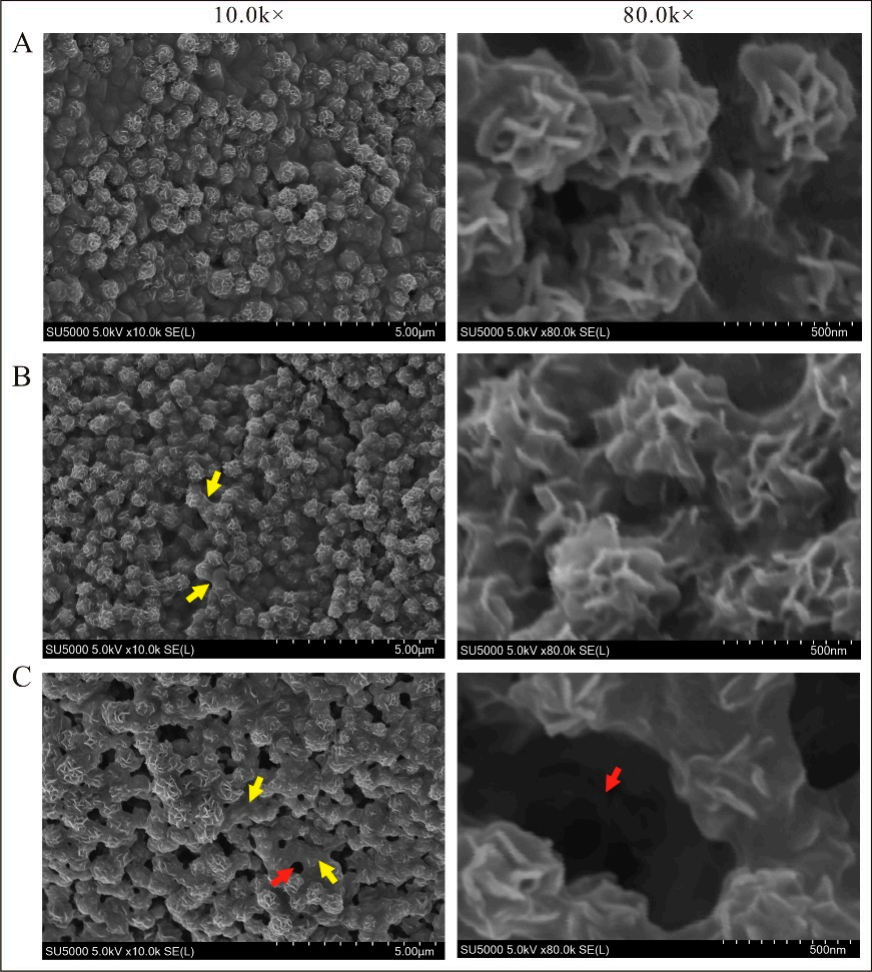
SEM characterizations of DNA hydrogels prepared using assembly of ssDNAs with different degrees of hydrogen bonding. (**A**): DNA hydrogel-1 prepared with two completely non-complementary DNA strands. (**B**): DNA hydrogel-2 prepared with two partially complementary ssDNA chains. (**C**): DNA hydrogel-3 prepared with two fully complementary DNA strands. Yellow arrows: locations where isolated nanoflower microstructures tended to connect with each other; red arrows: porous sheet structures.

The mechanical properties of hydrogels are closely correlated with their internal microstructure. Therefore, scanning electron microscopy (SEM) was employed next to observe the microstructure of the three types of prepared DNA hydrogels. Although all three exhibited a characteristic nanoflower microstructure (Figure 4A–C) comprised of RCA-generated ssDNA and had similar diameters (~500 nm) [8], the isolated nanoflower microstructures tended to connect with each other (yellow arrows, Figure 4B,C) with an increased degree of hydrogen bonding, gradually forming a porous sheet structure (red arrows, Figure 4C). This structure was similar to the microstructure of a DNA hydrogel produced with the hybridization chain reaction (HCR) technique [45,46], which can yield double helices [47]. The results intuitively demonstrate that as the degree of hydrogen bonding increased, the DNA cross-linking points became denser, and the internal microstructure of the gel became more complex.

**Scheme 2:**
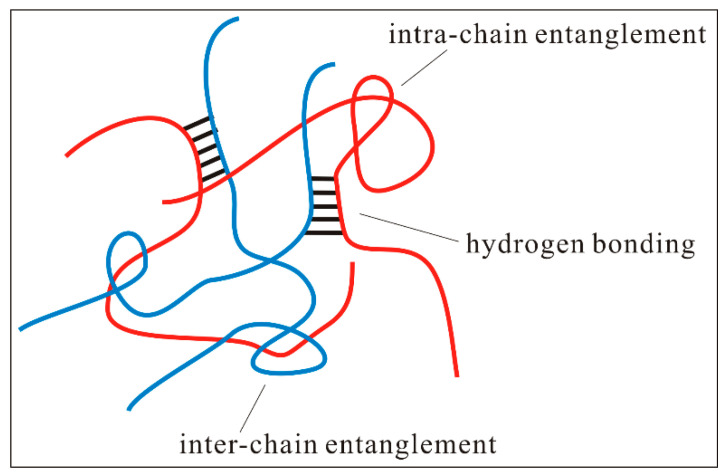
Schematic diagram of DNA hydrogels based on DNA chain entanglement (including intra- and inter-chain entanglement) and hydrogen bonding of long single-stranded DNAs.

Despite the complete complementarity of the ssDNA precursors in Figure 4C, the microstructure of the resulting nanoflowers was still observed. The incomplete hybridization of ssDNA by hydrogen bonding may account for this phenomenon. Other forces, such as hydrophilic–hydrophobic interaction, electrostatic interaction, and π–π stacking [15,48], contribute to the entanglement of ssDNA within and between strands (Scheme 2) [49]. From the preparation and characterization data of the aforementioned three gels, several conclusions can be inferred. For DNA hydrogel-1, the absence of hydrogen bond interactions between the two long DNA strands necessitates complete reliance on physical entanglement, including intra-chain and inter-chain physical entanglement, to form the gel. DNA hydrogel-2, on the other hand, is formed through a combination of hydrogen bond interactions and physical entanglement due to the presence of partially complementary bases between the two strands. Although the precursor DNA chains of DNA hydrogel-3 consist of completely complementary long ssDNA, it may not fully form dsDNA and retains some level of physical entanglement primarily influenced by hydrogen bond interactions. Non-covalent hydrogen bonds serve as network enhancers in DNA hydrogels, enhancing inter-chain interactions and promoting the mechanical strength and stability of the gel. Physical entanglement enables the gel to undergo sliding rather than fracturing when subjected to external forces, thereby contributing to the gel’s favorable tensile properties. Inter-chain entanglement may be more conducive to hydrogel stability and extensibility compared to intra-chain entanglement. In the absence of inter-chain entanglement, a distinct DNA hydrogel may not form or only a very loosely structured DNA hydrogel may be observed. This finding demonstrates the ability to finely adjust the mechanical properties of pure DNA hydrogels through precise control of factors such as the degree of base complementary pairing, hybridization temperature, hybridization time, oscillation time, and the method of regulating hydrogen bonding and physical entanglement. This level of control is important for facilitating specialized applications and plays a crucial role in the design of various functional DNA hydrogels.

### 3.5. Entrapment Efficiency Test of DNA Hydrogels

Because of their unique porous structure that can carry a large number of water-soluble compounds as well as the physical entanglement of DNA strands, DNA hydrogels have become a promising platform for encapsulating diverse particles and biomolecules in biosensing systems [50] and sustainable drug-delivery applications [51]. To further compare the entrapment efficiency of the hydrogels, we analyzed the ultraviolet absorption spectra of the varying amounts of gold nanoparticles (AuNPs) [52] present in the supernatant following gel entrapment. To prevent aggregation of the bare AuNPs in the salt-containing experimental system, we coated them with bovine serum albumin (BSA) before incorporating them into the hydrogel network. The AuNPs–BSA were then trapped inside the cross-linked network during hydrogel formation.

**Figure 5:**
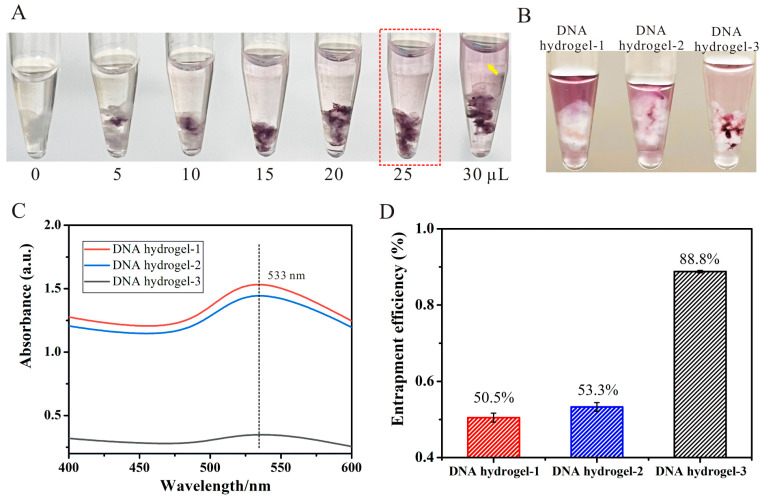
(**A**): Images of a series of different amounts of AuNPs–BSA (0, 5, 10, 15, 20, 25, 30 μL) trapped by DNA hydrogels prepared with full complementary ssDNA chains under identical conditions. (**B**): Images of the three kinds of DNA hydrogels loaded with 25 μL AuNPs–BSA. (**C**): UV–visible absorption spectra of three tubes of DNA hydrogels supernatant (AuNPs–BSA) in Figure B. The maximum absorption wavelength of the 30 nm AuNPs prepared in this study is 533 nm. (**D**): Corresponding entrapment efficiency of the three kinds of DNA hydrogels.

The entrapment efficiency of the DNA hydrogel based on full complementary ssDNA chains was initially assessed by examining the entrapment of varying amounts of AuNPs–BSA (0, 5, 10, 15, 20, 25, 30 μL) using seven hydrogels prepared under identical conditions. Figure 5A shows the results, whereby the supernatants of the tubes were transparent when the amount of AuNPs–BSA was less than 25 μL, indicating that most of the AuNPs were wrapped in the hydrogel. However, when the amount exceeded 25 μL, the supernatant of the tube turned red (yellow arrow, Figure 5A), indicating that a significant amount of free AuNPs–BSA remained. Therefore, 25 μL of AuNPs–BSA (dashed frame, Figure 5A) was chosen as the optimized indicator to test the entrapment ability of the different DNA hydrogels.

Three groups of DNA precursor solutions were supplemented with 25 μL of AuNPs–BSA and briefly vortexed to prepare hydrogels. As shown in Figure 5B, distinct hydrogels were formed in each of the three tubes and were observed to wrap a certain amount of AuNPs–BSA, as evidenced by the red color of the gels. UV absorption spectra were acquired from the supernatant of each tube, and the results are presented in Figure 5C. The three supernatants exhibited distinct UV–vis values at a wavelength of 533 nm, where 30-nm AuNPs have the maximum absorption. Of the three DNA hydrogels tested, DNA hydrogel-1 (based on completely non-complementary DNA strands) exhibited the highest absorbance, followed by DNA hydrogel-2 (based on partially complementary DNA strands), while DNA hydrogel-3 based on fully complementary DNA chains showed the lowest absorbance. Then, the entrapment efficiency of the DNA hydrogels for AuNPs–BSA was calculated using the following formula:

E = (A_total_ − A_hydrogel_)/A_total_;

where E represents the entrapment efficiency, A_total_ is the UV absorption value of 25 µL AuNPs–BSA which is shown in Figure S2, and A_hydrogel_ is the UV absorption value of the supernatant after entrapment of AuNPs–BSA with different hydrogels. After performing calculations, we determined that DNA hydrogel-3 exhibited the highest entrapment efficiency of 88.8%, followed by DNA hydrogel-2 at 53.3% and DNA hydrogel-1 at the lowest efficiency of 50.5% (Figure 5D). This result is consistent with the mechanical properties of the hydrogels, which suggests that in the presence of robust hydrogen bonding, the physical cross-linking force between the ssDNA precursor chains in DNA hydrogels is amplified, leading to the formation of a more condensed gel structure that is also capable of effectively incorporating more signaling molecules for biological detection or more drug molecules for efficient drug-delivery applications.

## 4. Conclusions

In summary, the dual rolling circle amplification (RCA) technique offers a promising strategy for producing physical pure DNA hydrogels with diverse mechanical properties. By utilizing ultralong ssDNA prepared with dual RCA, we synthesized three types of DNA hydrogels with varying degrees of hydrogen bonding comprising completely non-complementary, partially complementary, and fully complementary ssDNA chains. Our results demonstrate a close association between the performance of DNA hydrogels and the degree of hydrogen bonding, with an increase in hydrogen bonding degree leading to a corresponding enhancement of the gel’s mechanical strength and entrapment efficiency. Furthermore, we observed that large amounts of unrelated ssDNA precursors can also form hydrogel via intra- and inter-chain entanglement, although the mechanical properties of such gels are limited. Interestingly, even when using fully complementary DNA chains, the long single DNA chains tend to be partially wound rather than fully hybridized through base complementary hybridization. Compared to previous works, the advantages of using the dual RCA method for the preparation and regulation of DNA hydrogels are mainly as follows: (1). High efficiency. The long single-stranded DNA produced with RCA amplification can generate a large amount of DNA hydrogel by simply shaking at room temperature for 10 s. (2). Simple operation. The preparation process does not require any large instruments or complex operating steps. (3). Exclusion of other materials. By adjusting the sequence design of circular DNA, the mechanical properties of DNA hydrogels can be achieved, ensuring the excellent biocompatibility of the DNA hydrogel. At the same time, it should be noted that the mechanical properties of DNA hydrogels prepared with the current methods are still relatively low, and these hydrogels cannot be universally applied to demanding specialized fields. Additionally, the regulatory mechanisms still need further exploration, such as the proportion of hydrogen bonding interactions and physical entanglements, as well as the balance between intra-chain and inter-chain physical entanglements. The control of these proportions and their relationship with the performance of DNA hydrogels require further investigation. Additionally, the ratio of complementary sequences and the number of A–T and C–G base pairs may also affect the mechanical properties of the DNA hydrogel, which will be explored in our future studies.

## Figures and Tables

**Table 1:** DNA sequences used in this work.

DNA	Sequences (5′~3′)
Primer 1	CACAGCTGAGGATAGGACAT
Primer 2	GGACATGCAAGCAGAGCACA
Primer 3	ATGTCCTATCCTCAGCTGTG
PL-DNA-1	Phosphorylated-CTCAGCTGTGATTCATACGTACCAACGCACACAGAATT TTTTTTATGTCCTATC
PL-DNA-2	Phosphorylated-TTGCATGTCCAGTTCTTTGTCCTGAGTTTTACTGTGCCT GCTGCTGTGCTCTGC
PL-DNA-3	Phosphorylated-CTCAGCTGTGATTCATACGTTGGTACGCACACAGAATT TTTTTTATGTCCTATC
PL-DNA-4	Phosphorylated-GATAGGACATAAAAAAAATTCTGTGTGCGTTGGTACGT ATGAATCACAGCTGAG

The underlined bases are complementary; PL-DNA: phosphorylated linear DNA.

## Data Availability

Not applicable.

## Supplementary Materials

The following supporting information can be downloaded at: https://www.mdpi.com/article/10.3390/bios13070755/s1, Figure S1: Agarose gel electrophoresis results of the RCA product; Figure S2: UV–visible absorption spectra of AuNPs–BSA.
