# Supplementary material for: Investigation of the Impact of Hydrogen Bonding Degree in Long Single-Stranded DNA (ssDNA) Generated with Dual Rolling Circle Amplification (RCA) on the Preparation and Performance of DNA Hydrogels
Source: Biosensors (Basel). 2023 Jul 23;13(7):755. doi: 10.3390/bios13070755 (PMC10377478; doi:10.3390/bios13070755)
Supplement: Supplementary file 1 [file biosensors-13-00755-s001.zip › biosensors-2497439-supplementary.pdf]

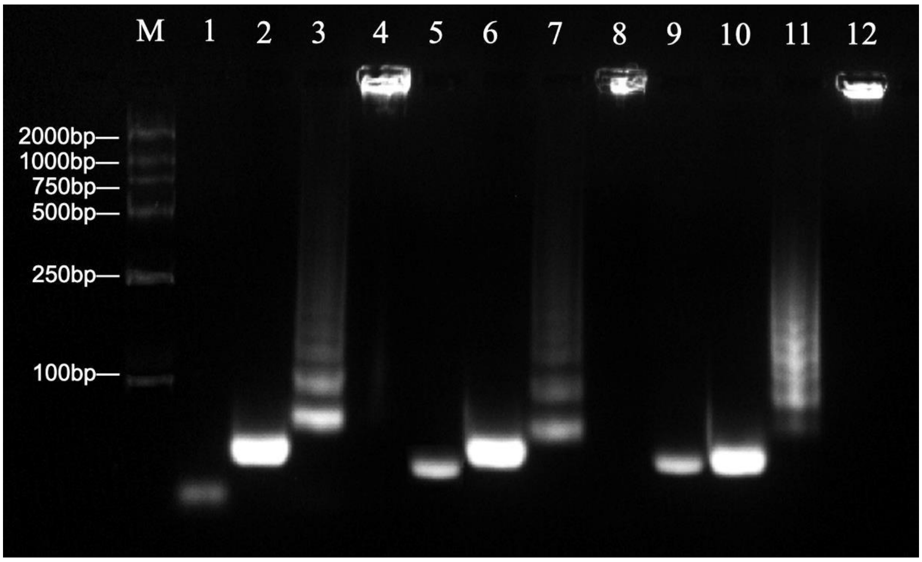

Figure S1. Agarose gel electrophoresis results of the RCA product. M: DNA marker; 1: primer-2; 2: phosphorylated linear DNA-2 (PL-DNA-2); 3: circular DNA template-2 (CT-2); 4: RCA products (ssDNA-2); 5: primer-3; 6: phosphorylated linear DNA-3 (PL-DNA-3); 7: circular DNA template-3 (CT-3); 8: RCA products (ssDNA-3); 9: primer-4; 10: phosphorylated linear DNA-4 (PL-DNA-4); 11: circular DNA template-4 (CT-4); 12: RCA products (ssDNA-4).

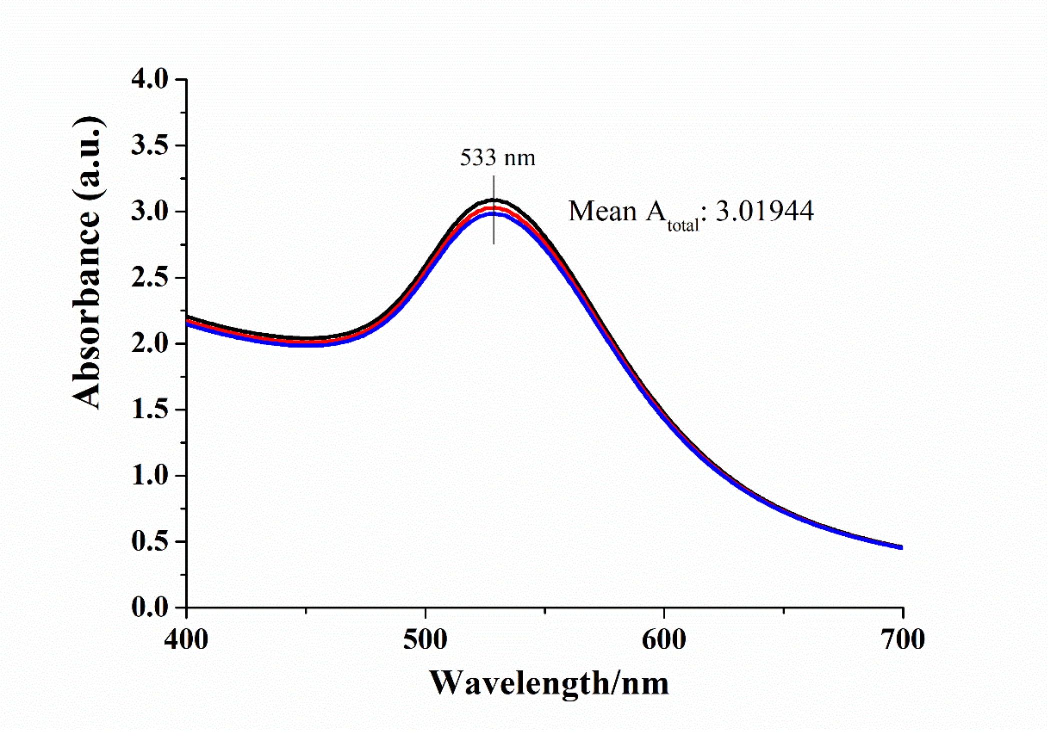

Figure S2. UV-visible absorption spectra of AuNPs-BSA. The maximum absorption wavelength of the 30 nm AuNPs prepared in this study is 533 nm. The average absorption value of the total amount of 25 μL of AuNPs-BSA was calculated to be 3.01944.
